# Structural insight into the arginine-binding specificity of CASTOR1 in amino acid-dependent mTORC1 signaling

**DOI:** 10.1038/celldisc.2016.35

**Published:** 2016-09-13

**Authors:** Jing Xia, Rong Wang, Tianlong Zhang, Jianping Ding

**Affiliations:** 1 School of Life Sciences, Shanghai University, Shanghai, China; 2 National Center for Protein Science Shanghai, State Key Laboratory of Molecular Biology, Institute of Biochemistry and Cell Biology, Shanghai Institutes for Biological Sciences, Chinese Academy of Sciences, Shanghai, China; 3 Shanghai Science Research Center, Chinese Academy of Sciences, Shanghai, China

**Keywords:** arginine, CASTOR1, crystal structure, GATOR complex, mTORC1 signaling

## Abstract

The mechanistic Target Of Rapamycin Complex 1 (mTORC1) is central to the cellular response to changes in nutrient signals such as amino acids. CASTOR1 is shown to be an arginine sensor, which plays an important role in the activation of the mTORC1 pathway. In the deficiency of arginine, CASTOR1 interacts with GATOR2, which together with GATOR1 and Rag GTPases controls the relocalization of mTORC1 to lysosomes. The binding of arginine to CASTOR1 disrupts its association with GATOR2 and hence activates the mTORC1 signaling. Here, we report the crystal structure of CASTOR1 in complex with arginine at 2.5 Å resolution. CASTOR1 comprises of four tandem ACT domains with an architecture resembling the C-terminal allosteric domains of aspartate kinases. ACT1 and ACT3 adopt the typical βαββαβ topology and function in dimerization via the conserved residues from helices α1 of ACT1 and α5 of ACT3; whereas ACT 2 and ACT4, both comprising of two non-sequential regions, assume the unusual ββαββα topology and contribute an arginine-binding pocket at the interface. The bound arginine makes a number of hydrogen-bonding interactions and extensive hydrophobic contacts with the surrounding residues of the binding pocket. The functional roles of the key residues are validated by mutagenesis and biochemical assays. Our structural and functional data together reveal the molecular basis for the arginine-binding specificity of CASTOR1 in the arginine-dependent activation of the mTORC1 signaling.

## Introduction

The mechanistic Target Of Rapamycin Complex 1 (mTORC1) is a key integrator of environmental conditions of nutrient, energy and extracellular signals such as insulin and growth factors [1, 2]. The aberrant activation of mTORC1 underlies the pathogenesis of many diseases, including cancer, neurodegeneration and diabetes [3, 4]. Among the wide range of signal inputs that impinge on the mTORC1 activity, amino acids are particularly potent activators upstream of mTORC1 in the anabolism and autophagy pathways [4–6]. With the availability of amino acids, mTORC1 is recruited to the lysosomal membrane through mediation of small GTPases RagA, -B, -C and -D, where its kinase activity is stimulated by the lysosome-anchored small GTPase Rheb [7, 8].

The small GTPase RagA or RagB forms a heterodimer with RagC or RagD, and the heterodimer is active when RagA/B is in the GTP-bound state and RagC/D in the GDP-bound state [8, 9]. The nucleotide-bound states of the Rag GTPases could be regulated by several proteins in response to amino acids. The Ragulator complex, a lysosome-anchored complex consisting of five subunits (LAMTOR1-5), functions as a scaffold for the Rag GTPases and additionally exerts guanine nucleotide-exchange factor activity towards RagA/B [8, 10]. The GATOR1 complex, consisting of three subunits (NPRL2, NPRL3 and DEPDC5), functions as a GTPase activating protein (GAP) for RagA/B, whereas the GATOR2 complex, consisting of five subunits (WDR59, WDR24, MIOS, SEH1L and SEC13), acts as an inhibitor of GATOR1 [11]. In addition, the FLCN-FNIP2 complex serves as a GAP for RagC/D in an amino acid-sensitive fashion [12]. However, the molecular mechanisms by which amino acid signals are transduced to these protein complexes and promote the activation or inhibition of the Rag GTPases and mTORC1 are still elusive.

Very recently, several amino acid sensing proteins are reported to function as upstream regulators of the Rag GTPases and the mTORC1 pathway. The leucine sensor Sestrin2 can interact with GATOR2 and disrupts the GATOR1–GATOR2 interaction and then releases the GAP activity of GATOR1 for the Rag GTPases; and the binding of leucine to Sestrin2 blocks its interaction with GATOR2, leading to the inhibition of GATOR1 [13, 14]. In addition to leucine, arginine is also shown to be an important activator of the mTORC1 pathway [15, 16]. The amino acid transporter SLC38A9 is identified to be a lysosome-based arginine sensor for mTORC1 [17, 18], and the ACT [Pfam 01842; a small regulatory domain found in Aspartate kinase, Chorismate mutase and TyrA (prephenate dehydrogenase)] domain-containing protein CASTOR1 is a cytoplasm-localized arginine sensor [19]. Similar to the regulation mechanism of Sestrin2 with GATOR2, CASTOR1 can also inhibit the GATOR2–GATOR1 mediated mTORC1 activation in starving cells; and the binding of arginine to CASTOR1 disrupts the CASTOR1–GATOR2 interaction and hence GATOR2 is released to form the GATOR1–GATOR2 complex, leading to the inhibition of GATOR1 [19]. To understand the molecular mechanism of the arginine-binding specificity of CASTOR1, we determine the crystal structure of CASTOR1 in complex with arginine. The structural data together with biochemical data reveal the molecular basis for the arginine-binding specificity of CASTOR1 in the arginine-dependent activation of the mTORC1 signaling.

## Results

### Structure of CASTOR1

The full-length human CASTOR1 (residues 1–329) was successfully expressed in and purified from *E. coli* (Supplementary Figure S1A). The recombinant CASTOR1 protein exists as a homodimer in solution as revealed by gel filtration and dynamic light scattering analyses, and the dimerization is independent of arginine (Supplementary Figure S1B and C). Attempts to crystallize CASTOR1 alone were unsuccessful; however, crystallization of CASTOR1 in the presence of arginine yielded crystals of arginine-bound CASTOR1. The structure of the arginine-bound CASTOR1 was solved by the single-wavelength anomalous dispersion method and was refined to 2.5 Å resolution, yielding an R-factor of 17.5% and a free R-factor of 22.4% (Table 1). There are four arginine-bound CASTOR1 molecules in an asymmetric unit, forming two CASTOR1 homodimers which have almost identical overall structure with a root-mean-square deviation of 0.9 Å for 570 Cα atoms (Supplementary Figure S2A and B). In the following structural analysis and discussion, molecule A is used as the representative, in which most residues of the polypeptide chain are well defined with high-quality electron density except for a few surface exposed loops (residues 86–89, 158–165, 214–217 and 325–329; Supplementary Figure S3).

**Figure 1:**
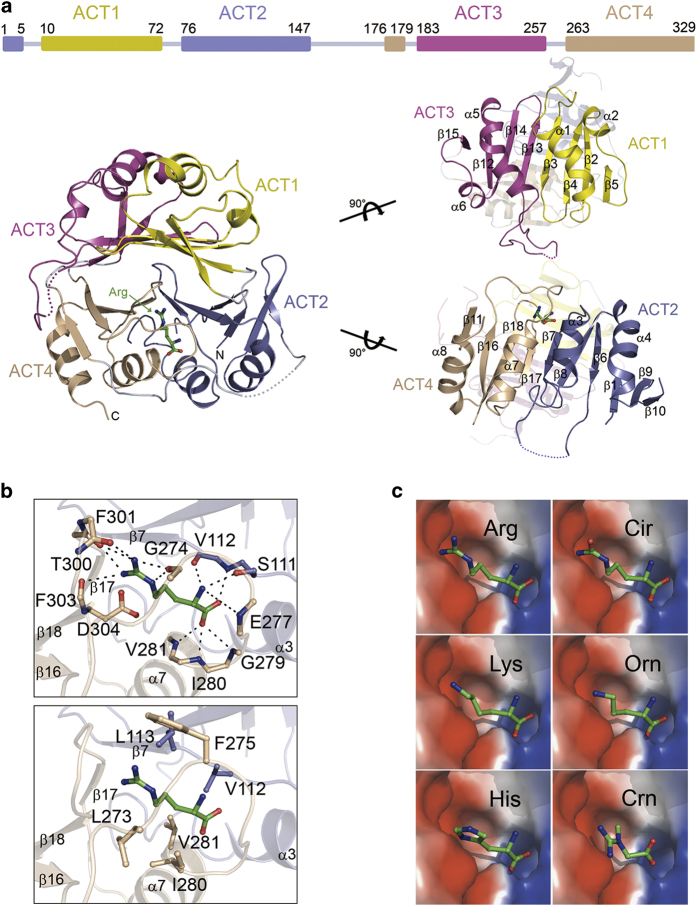
Crystal structure of CASTOR1 in complex with arginine. (**a**) Overall structure of CASTOR1 in complex with arginine. The ACT1–4 domains are colored in yellow, blue, violet and wheat, respectively. The bound arginine is shown with a stick model in green. (**b**) Structure of the arginine-binding site. Upper panel: hydrogen bonds and salt bridge interaction of arginine with the surrounding residues. Lower panel: hydrophobic interactions of arginine with the surrounding residues. (**c**) Electrostatic surface of the arginine-binding site to show the fitness of arginine (Arg) and several other amino acids including lysine (Lys), histidine (His), citrulline (Cir), ornithine (Orn) and creatine (Crn) in the arginine-binding pocket. The other amino acids are modeled into the arginine-binding pocket based on the positions of the main-chain atoms.

Previously it was suggested based on sequence analysis that CASTOR1 is consisted of two tandem ACT domains [19]. The structure of CASTOR1 shows it composed of four tandem ACT domains (ACT1-4; Figure 1a). ACT1 (residues 10–72) and ACT3 (residues 183–257) adopt the typical βαββαβ topology of the ACT domain [20]. ACT 2 and ACT4 both comprise of two non-sequential regions (residues 1–5 and 76–147, and residues 176–179 and 263–329, respectively) and exhibit the unusual ββαββα topology of the ACT domain [21] with their first β-strand located before ACT1 and ACT3, respectively; and additionally, ACT2 contains two extra β-strands (strand β9 and β10) at the C-terminal, thus forming a six-stranded β-sheet instead of a four-stranded β-sheet observed in the other ACT domains (Supplementary Figure S4A and B).

ACT1 and ACT2 make up the N-terminal region and ACT3 and ACT4 the C-terminal region (Supplementary Figure S4A and B). The two ACT domains in the N-terminal region and C-terminal region are orthogonal to each other, and the two regions have a high structural similarity with a root-mean-square deviation of 1.8 Å for 116 Cα atoms with 25% sequence identity (Supplementary Figure S4C). However, ACT1 and ACT3 fold together to form an 8-stranded β-sheet flanked by four α-helices on one side, and ACT2 and ACT4 fold together to form a 10-stranded β-sheet flanked by four α-helices on the other side, and the two β-sheets are orthogonal to each other and form a β-barrel-like core which is sandwiched by the two layers of α-helices (Figure 1a). The previous co-immunoprecipitation (co-IP) analyses revealed that the divided N-terminal region and C-terminal region interact with each other only in the presence of arginine [19], suggesting that the binding of arginine at the interface of ACT2 and ACT4 is essential for the interaction between these two domains. It is possible that the absence of arginine may cause the dissociation and rearrangement of ACT2 and ACT4 and thus creates a binding site on CASTOR1 for GATOR2.

In the structure of CASTOR1, two CASTOR1 molecules form a tight homodimer via a twofold non-crystallographic symmetry. The homodimer interface is stabilized by largely hydrophobic interactions and a few hydrogen-bonding interactions among residues from helices α1 of ACT1 and α5 of ACT3 of each monomer (Supplementary Figure S5A and B), which buries 1 857 Å^2^ or 7.1% of the total solvent accessible surface area as analyzed by the PISA server [22]. Sequence alignment shows that the residues involved in the dimerization are strictly conserved in different species (Supplementary Figure S6).

### Arginine-binding site

In the structure of CASTOR1, arginine binds to the interface of the ACT2 and ACT4 domains with well-defined electron density and reasonable B-factor. The arginine-binding pocket is largely formed by residues from α7, the β17-β18 loop and β18 of ACT4 and the α3-β7 loop of ACT2, and additionally the β16-α7 loop (residues 269–279) acts as a lid to conceal the binding pocket (Figure 1b). Several residues of these structural elements make a number of hydrogen-bonding interactions and extensive hydrophobic contacts with arginine (Figure 1b). In detail, the α-carboxyl group of arginine forms four hydrogen bonds with the main chains of Val112, Gly279, Ile280 and Val281; and the α-amino group forms three hydrogen bonds with the side chain of Ser111 and the main chains of Val112 and Glu277. The side chain of arginine points toward the β17-β18 loop. The guanidinium group forms five hydrogen bonds with the main chains of Gly274, Thr300, Phe301 and Phe303, and additionally makes a salt bridge with the side chain of Asp304. Moreover, the aliphatic side chain of arginine is further stabilized by extensive hydrophobic interactions with the side chains of Val112, Leu113, Leu273, Ile280 and Val281. Sequence alignment shows that most of the residues involved in the arginine binding are highly conserved in different species (Supplementary Figure S6).

**Figure 2:**
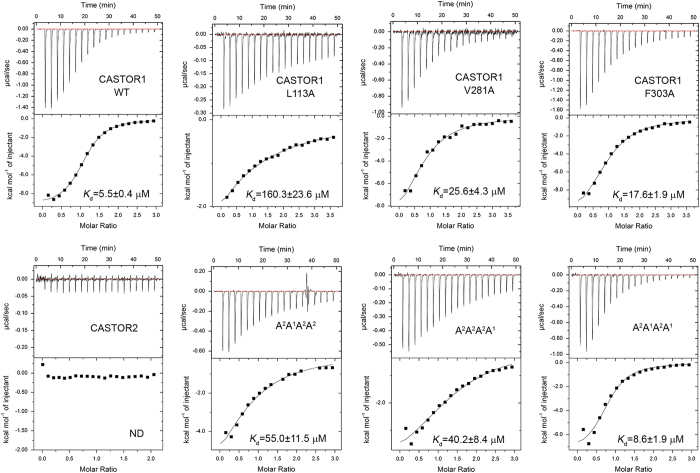
Isothermal titration calorimetry measurements for the arginine-binding affinity of the wild-type and mutant CASTOR1 and CASTOR2, and the three chimeric proteins of A^2^A^1^A^2^A^2^, A^2^A^1^A^2^A^1^ and A^2^A^2^A^2^A^1^. ND, not detected.

To validate the functional roles of these residues, we performed mutagenesis and isothermal titration calorimetry (ITC) analyses. Our ITC assay results show that the wild-type CASTOR1 binds arginine with a dissociation constant (*K*_d_) of 5.5±0.4 μM (Table 2 and Figure 2), which is lower than that obtained from the equilibrium binding assay (~35 μM) [19]. The S111A (or S111L) and D304L mutants have no measurable *K*_d_, indicating that these mutations disrupt the arginine binding (Table 2). The L113A, V281A and F303A mutants have substantially increased *K*_d_, and the L273A and I280A mutants have no measurable *K*_d_, indicating that these mutations also dramatically impair the arginine binding of CASTOR1 (Table 2 and Figure 2). These results demonstrate that Ser111 and Asp304 play a critical role in the arginine binding and the residues involved in the hydrophobic contacts also play an important role in the binding. Intriguingly, Phe275 of the β16-α7 loop has no direct interaction with arginine (Figure 1b), however, mutation F275A diminishes the arginine binding of CASTOR1 (Table 2), indicating that Phe275 also plays an important role in the arginine binding probably through stabilizing the conformation of the β16-α7 loop and hence the arginine-binding pocket.

Our ITC assays also show that CASTOR1 displays a high-binding affinity for arginine, but has no measurable binding for other amino acids including leucine, lysine and histidine (Table 2). Arginine is a non-essential amino acid for adults, which can be synthesized from glutamate with ornithine and citrulline as intermediate products. Ornithine has a positively charged side chain as arginine with an amino group substituting the guanidinium group, and citrulline has a similar side chain as arginine with a carbonyl group substituting one η-amino group of the guanidinium group. Creatine is naturally produced from arginine and glycine in cells. Thus, we also tested the binding ability of CASTOR1 with these amino acids and our ITC assays show that CASTOR1 has no measurable binding for ornithine, citrulline and creatine (Table 2). These results can be explained very well by the structure of CASTOR1. The bound arginine makes extensive hydrophilic and hydrophobic interactions with the surrounding residues composing the arginine-binding pocket. Particularly the guanidinium group is embedded in an acidic pocket formed by the side chain of Asp304 and the main-chain carbonyls of Gly274, Thr300, Phe301 and Phe303, and makes a series of hydrogen-bonding interactions with these residues (Figure 1b). Thus, any residue with a shorter or/and a differently charged side chain could not maintain these interactions and thus would not be able to bind to CASTOR1 (Figure 1c). This provides the molecular basis for the high-binding specificity of CASTOR1 for arginine.

### Difference in the arginine sensitivity between CASTOR1 and CASTOR2

In human, there are two CASTOR proteins, namely CASTOR1 and CASTOR2, which share ~63% sequence identity. The co-IP experiments showed that CASTOR1 and CASTOR2 could interact with GATOR2 probably at the same binding site [19]. Like CASTOR1, CASTOR2 also exists as a homodimer in solution (Supplementary Figure S1B and C). The previous co-IP experiments also showed that CASTOR2 could form a complex with CASTOR1 [19]. Intriguingly, our ITC assay results show that there is no evident interaction between the recombinant CASTOR1 and CASTOR2 homodimers (data not shown); however, we were able to obtain the CASTOR1–CASTOR2 complex using the co-expression and co-purification methods (Supplementary Figure S1A), which exists as a heterodimer in solution (Supplementary Figure S1B and C). As the residues involved in the dimerization of CASTOR1 are highly conserved in CASTOR2 (Supplementary Figure S7), it is possible that the CASTOR2 homodimer and the CASTOR1–CASTOR2 heterodimer might assume similar assembly as the CASTOR1 homodimer. In addition, it seems that the CASTOR1 and CASTOR2 homodimers are sufficient stable, and they cannot dissociate each other to form the CASTOR1–CASTOR2 heterodimer *in vitro*.

Unlike CASTOR1, CASTOR2 interacts with GATOR2 in an amino acid insensitive manner [19]. This is also confirmed by our ITC assay showing that CASTOR2 has no detectable binding with arginine (Table 2 and Figure 2). Intriguingly, the majority of residues composing the arginine-binding pocket of CASTOR1 are strictly conserved in CASTOR2 except for Leu113, which is replaced by Phe115 in CASTOR2 (Supplementary Figure S7). However, mutation F115L in CASTOR2 does not confer an arginine sensitivity (data not shown). Thus, the difference in the arginine sensitivity between CASTOR1 and CASTOR2 should be attributed to the residues outside the arginine-binding pocket and/or some other factors. For example, as the arginine-binding pocket is located in the interface of ACT2 and ACT4, the two ACT domains might assume different conformations in CASTOR1 and CASTOR2, thus resulting a differed binding ability with arginine. To explore this possibility, we constructed three chimeric proteins of CASTOR2 in which ACT2 or/and ACT4 were substituted with those of CASTOR1. The chimeric CASTOR2 containing ACT2 of CASTOR1 (A^2^A^1^A^2^A^2^) exhibits a *K*_d_ of 55.0±11.5 μM for arginine, the chimeric CASTOR2 containing ACT4 of CASTOR1 (A^2^A^2^A^2^A^1^) exhibits a *K*_d_ of 40.2±8.4 μM, and the chimeric CASTOR2 containing both ACT2 and ACT4 of CASTOR1 (A^2^A^1^A^2^A^1^) has a higher affinity of 8.6±1.9 μM which is comparable to that of CASTOR1 (Table 2 and Figure 2). These results suggest that both ACT2 and ACT4 of CASTOR2 contribute to its inability for arginine binding.

## Discussion

**Figure 3:**
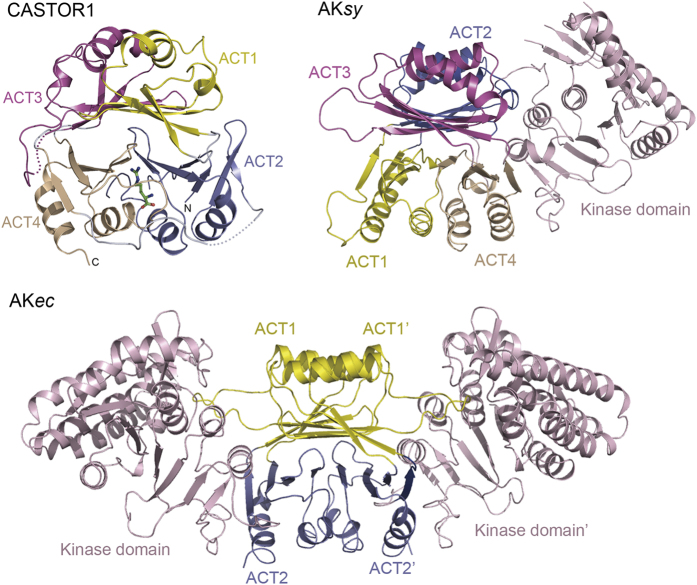
Structural comparison of CASTOR1 and aspartate kinases from *Cyanobacteria Syncchocystis* (AK*sy*; PDB code 3l76) and *E. coli* (AKec; PDB code 2J0W). The ACT1–4 domains are colored as Figure 1.

Using the Dali server to carry out the structural similarity search in the Protein Data Bank [23], we found that the overall architecture of CASTOR1 resembles those found in many aspartate kinases from archaebacteria, cyanobacteria, actinobacteria, proteobacteria and viridiplantae, which contain two or four ACT domains in the C-terminal region to allosterically regulate the N-terminal kinase domain (Figure 3) [24]. The typical aspartate kinase from *E. coli* (AK*ec*) is homodimeric which is formed between the ACT domains from two neighboring subunits (Figure 3). In particular, CASTOR1 is structurally very similar to the Cyanobacteria Synchocystis aspartate kinase (AK*sy*) which contains four ACT domains in the C-terminal region but with different ACT domain organization (Figure 3). Aspartate kinases catalyze the phosphorylation of aspartate in the biosynthesis of lysine, threonine, methione and isoleucine [24], but is deficient in human. It is possible that CASTOR1, which is only found in several vertebrates, was evolved from the ancient aspartate kinase but lost the N-terminal kinase domain during the evolution. ACT domains are well known regulatory domains in many allosteric enzymes. CASTOR1 may possess a similar allosteric regulatory activity for the enzymes in arginine synthesis. If so, CASTOR1 forms a complex with GATOR2 and hence inhibits the mTORC1 signaling in arginine starved cells, while the arginine-bound CASTOR1 may dissociate from the CASTOR1-GATOR2 complex and inhibits the arginine synthesis in the cells. Further work might be worthwhile to explore the potential functions of CASTOR1 in amino acid metabolism.

During the preparation of this manuscript, a study from the Sabatini group reported the crystal structure of CASTOR1 in complex with arginine [25]. The overall structure of CASTOR1 and particularly the arginine-binding site from both studies are very similar. There are only a few minor differences in the definition of secondary structures of ACT3 and ACT4 as two extra short β-strands are defined in our structure and the corresponding regions are disordered in the structure of Saxton *et al.* [25]. In Saxton *et al.* [25], the authors have validated the functional roles of the key residues at the arginine-binding site and the homodimer interface using both *in vitro* (arginine-binding assay) and *in vivo* (co-IP and mTORC1 activity assay) functional analyses [25]. Our *in vitro* functional assay results are consistent with and complement to Saxton *et al.* [25]. These two studies together reveal the molecular basis for the unique binding specificity of CASTOR1 for arginine, and provide insight into the molecular mechanism of arginine sensing by CASTOR1 upstream of the mTORC1 pathway.

## Materials and Methods

### Cloning, expression and purification

The gene encoding the human full-length CASTOR1 was inserted into the *Nco*I and *Xho*I restriction sites of the pET-28a plasmid (Novagen, Madison, WI, USA) with a His_6_-tag attached at the C-terminus of the target protein. The plasmid was transformed into *E. coli* BL21 (DE3) strain, and the transformed cells were grown at 37 °C in lysogeny broth medium containing 0.05 mg ml^−1^ kanamycin until OD_600_ reached 0.8, and then induced with 0.5 mM isopropyl-β-D-thiogalactopyranoside (IPTG) at 20 °C for 20 h. The cells were harvested and lysed by sonication in lysis buffer (30 mM Tris-HCl, pH 7.5 and 200 mM NaCl). The His_6_-tagged CASTOR1 was purified by affinity chromatography using a Ni-NTA column (Qiagen, Hilden, Germany) with the lysis buffer supplemented with 30 mM imidazole and 200 mM imidazole serving as washing buffer and elution buffer, respectively, and further purified by gel filtration using a Superdex 200 10/300 column (GE Healthcare, Little Chalfont, UK). The purified protein, which was of sufficient purity (>95%) as determined by sodium dodecyl sulfate polyacrylamide gel electrophoresis (12% gel), was stored in the buffer consisting of 10 mM HEPES, pH 7.5, 100 mM NaCl, and 1 mM DTT.

To obtain the SUMO fusion proteins of CASTOR2, the genes were inserted into a modified pET-28a vector, which attaches a His_6_-SUMO tag at the N-terminal of the target protein. To obtain the CASTOR1–CASTOR2 complex, *castor1* was inserted into the pET-Duet plasmid (Novagen) without tag and co-transformed into *E. coli* BL21 (DE3) strain with the His_6_-SUMO fused *castor2* plasmid. Constructs of the *castor1* and *castor2* mutants containing point mutations or substitutions were generated using the QuikChange Site-Directed Mutagenesis Kit (Strategene, La Jolla, CA, USA) following the instruction manuals. Expression and purification of these proteins were the same as described above. The N-terminal His_6_-SUMO tag of the purified proteins could be removed by an ubiquitin-like protease (ULP1).

### Crystallization, data collection and structure determination

Crystallization of CASTOR1 was performed using the hanging drop vapor diffusion method by mixing 1 μl protein solution (~10 mg ml^−1^) supplemented with 5 mM arginine and 1 μl reservoir solution at 16 °C. Crystals were grown from drops consisting of a reservoir solution of 0.1 M sodium citrate (pH 5.0) and 20% (w/v) polyethylene glycol 8 000 (PEG8000). Diffraction data from crystals of Se-Met substituted and native proteins were collected at −175 °C at beamline 19U1 of National Facility for Protein Science in Shanghai, China, and were processed with HKL3000 [26]. Statistics of the diffraction data are summarized in Table 1.

The structure of CASTOR1 was solved using the single-wavelength anomalous dispersion method implemented in Phenix [27], which yielded an overall figure of merit (FOM) of 0.30 and identified all 20 Se sites of four CASTOR1 monomers in an asymmetric unit. Initial structure model was built by the AutoBuild program in Phenix [27], and the final structure model was built manually using Coot [28]. Structure refinement was carried out using Phenix and Refmac5 [27, 29]. Stereochemistry and quality of the structure model were analyzed using programs in CCP4 [30]. Structure figures were prepared using Pymol (http://www.pymol.org). Statistics of the structure refinement and the quality of the structure model are also summarized in Table 1.

### Isothermal titration calorimetry analysis

Isothermal titration calorimetry (ITC) measurements were performed at 20 °C using an ITC200 Micro-Calorimeter (Malvern, Worcestershire, UK). An initial injection of 0.2 μl protein was discarded for each data set to remove the effect of titrant diffusion across the syringe tip during the equilibration process. For Castor1/Castor2 (wild-type, mutants and variants), each experiment consisted of 20 injections of 2 μl arginine (or other amino acids, 1 mM) into the sample cell containing 290 μl protein (70 μM). A background titration was performed using identical titrant with the buffer solution placed in the sample cell. Titration curves were fit by a nonlinear least-squares method implemented in MicroCal Origin software version 7.0 using the single binding site model.

### Accession code

The structure of CASTOR1 in complex with arginine has been deposited in the RCSB Protein Data Bank with accession code 5GS9.

## Figures and Tables

**Table 1:** Summary of X-ray diffraction data and structure refinement statistics

	*Se-Met*	*Native*
*Data collection*		
Wavelength (Å)	0.9785	0.9785
Space group	*P*2_1_	*P*2_1_
Resolution (Å)	50.00–2.80 (2.90–2.80)^a^	50.00–2.50 (2.59–2.50)
Cell parameters		
* * *a, b, c* (Å)	93.9, 82.8, 98.2	93.6, 83.6, 97.8
* * β (°)	116.6	116.6
Observed reflections	460 764	317 238
Unique reflections (I/*σ* (I)>0)	33 667	47 062
Average redundancy	13.7 (13.2)	6.7 (6.2)
Average I/*σ* (I)	19.6 (3.7)	15.9 (2.5)
Completeness (%)	100.0 (99.9)	99.9 (97.9)
Rmerge (%)	14.6 (66.2)	8.5 (49.5)
		
*Refinement*		
Reflections (Fo≥0 *σ*(Fo))		
Working set/test set		42 055/2 360
*R*_work_/*R*_free_		0.175/0.224
No. of protein atoms		9 249
No. of arginine atoms		48
No. of water atoms		314
Average B factor of all atoms (Å^2^)		52.2
Protein atoms		52.4
Arginine atoms		42.6
Water atoms		47.5
r.m.s.d.		
Bond lengths (Å)		0.007
Bond angles (°)		1.25
Ramachandran plot (%)		
Favored		98.8
Allowed		1.2
Outliers		0

Abbreviation: r.m.s.d., root-mean-square deviation.

a Numbers in parentheses represent the highest resolution shell.

**Table 2:** ITC measured thermodynamic parameters

	K*_d_ (μM)*	*ΔH (kcal mol^−1^)*	*TΔS (kcal mol^−1^)*	n*-value*
*CASTOR1 with ligands*				
Arginine	5.5±0.4	−9.3±0.1	−2.29	1.09±0.01
Lysine	ND	ND	ND	ND
Histidine	ND	ND	ND	ND
Citrulline	ND	ND	ND	ND
Ornithine	ND	ND	ND	ND
Creatine	ND	ND	ND	ND
				
*CASTOR1 mutants with arginine*				
S111A	ND	ND	ND	ND
S111L	ND	ND	ND	ND
L113A	160.3±23.6	−11.3±6.7	−6.18	0.56±0.30
L273A	ND	ND	ND	ND
F275A	ND	ND	ND	ND
I280A	ND	ND	ND	ND
V281A	25.6±4.3	−11.8±1.6	−5.60	0.79±0.08
F303A	17.6±1.9	−12.1±0.61	−5.77	0.96±0.04
D304L	ND	ND	ND	ND
				
*CASTOR2 and the chimeric proteins with arginine*				
CASTOR2	ND	ND	ND	ND
A^2^A^1^A^2^A^2^	55.0±11.5	−9.4±2.4	−3.69	0.77±0.15
A^2^A^2^A^2^A^1^	40.2±8.4	−4.8±0.6	1.10	1.45±0.10
A^2^A^1^A^2^A^1^	8.6±1.9	−7.6±0.6	−0.83	0.79±0.05

Abbreviations: ITC, Isothermal titration calorimetry; *K*_d_, dissociation constant; ND, not detected.
