# Supplementary material for: Structural insight into the arginine-binding specificity of CASTOR1 in amino acid-dependent mTORC1 signaling
Source: Cell Discov. 2016 Sep 13;2:16035–. doi: 10.1038/celldisc.2016.35 (PMC5020642; doi:10.1038/celldisc.2016.35)

Supplementary Materials

Supplementary Figure legends

Figure S1. Analyses of the oligomeric states of CASTOR1, CASTOR2 and the CASTOR1-CASTOR2 complex in solution. (A) SDS analyses of the purified CASTOR1-His$_6$, His$_6$-SUMO-CASTOR2, and His$_6$-SUMO-CASTOR2-CASTOR1 complex by affinity chromatography using a Ni-NTA column. The N-terminal His$_6$-SUMO tag of CASTOR2 was removed by ULP1. (B) Size-exclusion chromatography of CASTOR1, CASTOR2, CASTOR1-CASTOR2 on Superdex 200 10/300 column. All samples were injected into the column with 500 μL. (C) Dynamic light scattering (DLS) analysis of CASTOR1, CASTOR2, CASTOR1-CASTOR2. DLS analysis was performed using a DynaPro Tytan instrument equipped with a temperature-controlled MicroSampler (Wyatt Technology) at a laser wavelength of 662.30 nm at 25 ℃. Each measurement consisted of fifties 5s acquisitions. All samples were prepared with 100 μM. To obtain the hydrodynamic radii (Rh) and polydispersity, the intensity autocorrelation functions were fitted with a non-negative least-squares algorithm using Dynamics 7.1.7.16 software (Wyatt Technology).

Figure S2. Architecture of CASTOR1 in an asymmetric unit. (A) Ribbon representation of the two CASTOR1 homodimers (colored in pink and yellow, respectively) in the asymmetric unit. The bound arginine is shown with a green stick model. (B) Superposition of the two CASTOR1 homodimers in the asymmetric unit.

Figure S3. Composite simulated annealing 2Fo-Fc omit map (contoured at 1.5σ) for arginine and the two critical residues of Ser111 and Asp304.

Figure S4. Ribbon representation and topology of secondary structure elements of CASTOR1. (A) NTR of CASTOR1. (B) CTR of CASTOR1. (C) Superposition and sequence alignment of NTR and CTR.

Figure S5. Dimer interface of the CASTOR1 homodimer. (A) Ribbon representation of the CASTOR1 homodimer. The ACT domains are colored the same as Figure 1. (B) View of the residues involved in the dimer interface.

Figure S6. Sequence comparison of CASTOR1 from different species. The secondary structure elements of human CASTOR1 are placed on the top of the alignment. The residues that are involved in arginine binding are indicated with triangles. The sequence alignment was performed using the program ESPript [1].

Figure S7. Sequence comparison of CASTOR1 and CASTOR2. The residues that are involved in arginine binding are indicated with triangles while the residues involved in the dimer interface are marked with stars. The sequence alignment was performed using the program ESPript [1].

Reference

1 Gouet P, Courcelle E, Stuart DI, Metoz F. ESPript: analysis of multiple sequence alignments in PostScript. *Bioinformatics (Oxford, England)* 1999; **15**:305-308.

Figure S1

A

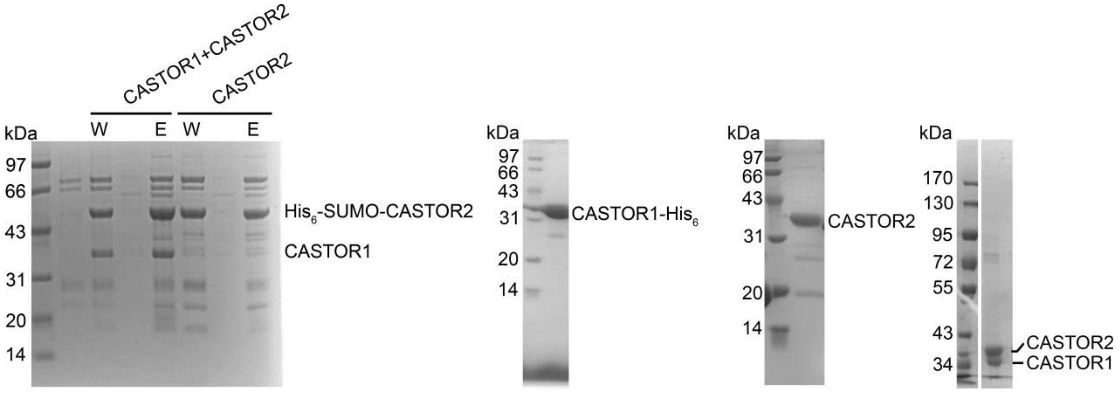

B

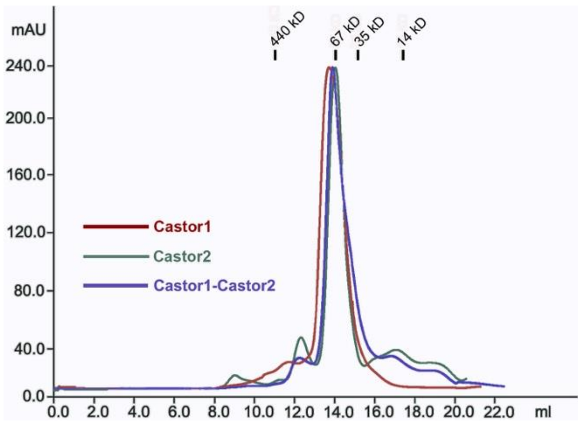

C

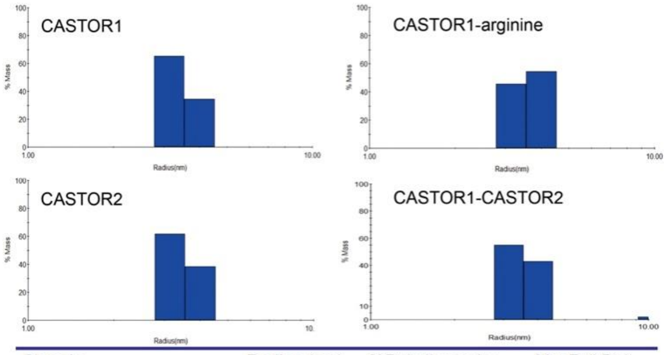

Sample	Radius (nm)	%Polydispersity	Mw-R (kDa)
CASTOR1	3.446	12.1	61
CASTOR1-arginine	3.620	12.1	68
CASTOR2	3.481	12.3	62
CASTOR1-CASTOR2	3.528	12.3	64

Mw-R is the estimated mass based on the determined hydrodynamic radius and particle density for the globular protein model.

Figure S2

A

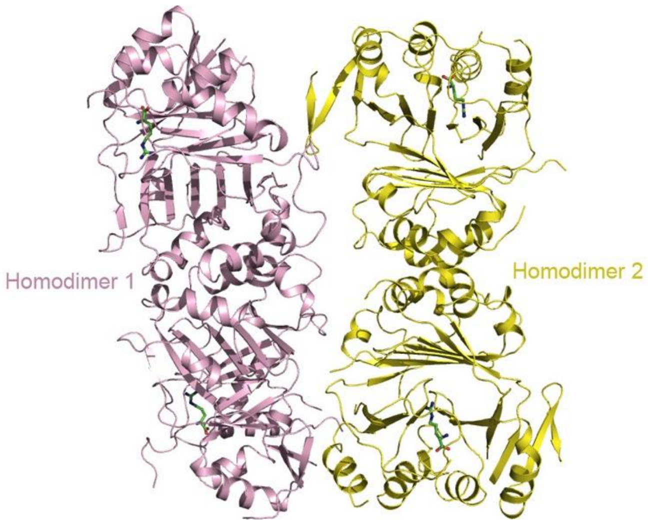

B

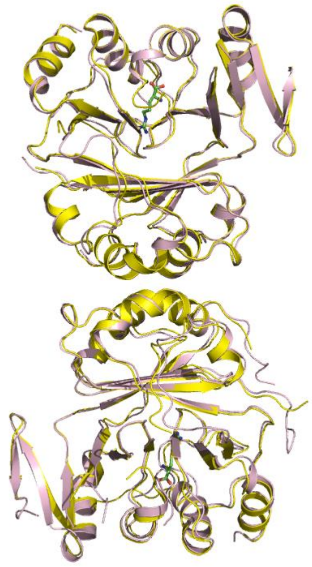

Figure S3

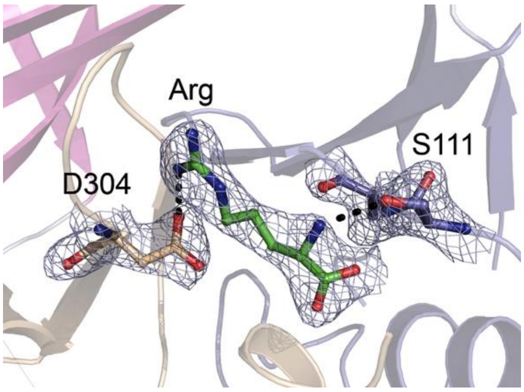

Figure S4

A

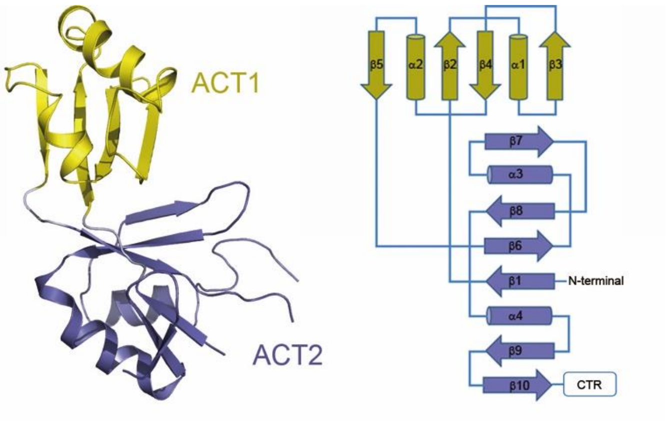

B

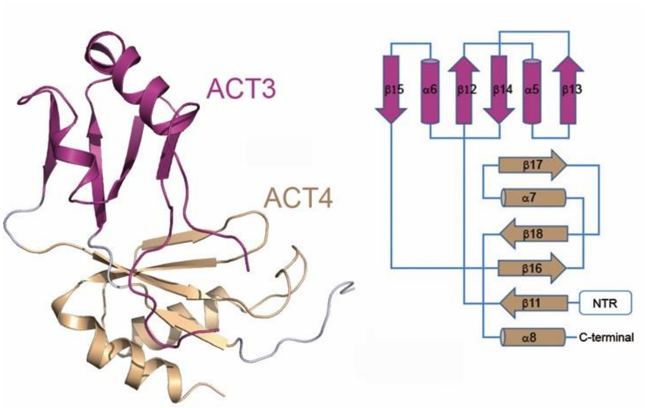

C

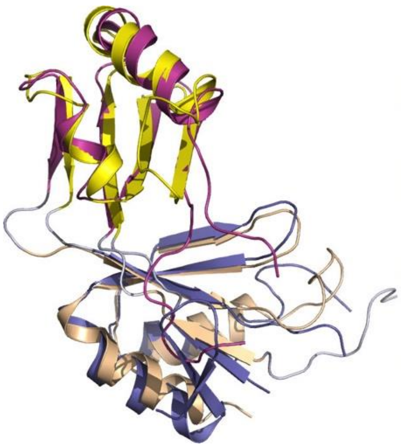

```
                1         10        20        30
NTR   ..........MELHILEHRVRVLSVARPGLWLYTHPLIKLLFL.......
CTR   RTQHGPSPTVHPIQSPQNRFCVLTLDPETLPAIATTLIDVLFYSHSTPKE

                40        50        60        70
NTR   .....PRRSRCKFFSLTETPEDYTLMVDEEGFKELPPSEFLQVAEA.TWL
CTR   AASSSPEPSSITFFAFSLIEGYISIVMDAETQKKFPSDLLLTSSSGELWR

        80        90        100       110       120
NTR   VLNVSSHSGAAVQAAGVTKIARSVIAPLAEHHVSVLMLSTYQTDFILVRE
CTR   MVRIGGQPLGFDECGIVAQIAG....PLAAADISAYYISTFNFDHALVPE

      130       140       150       160
NTR   QDLSVVIHTLAQEFDIYREVGGEPVPVTRDDSSNGFP
CTR   DGIGSVIEVLQRRQEGLAS..................
```

Figure S5

A

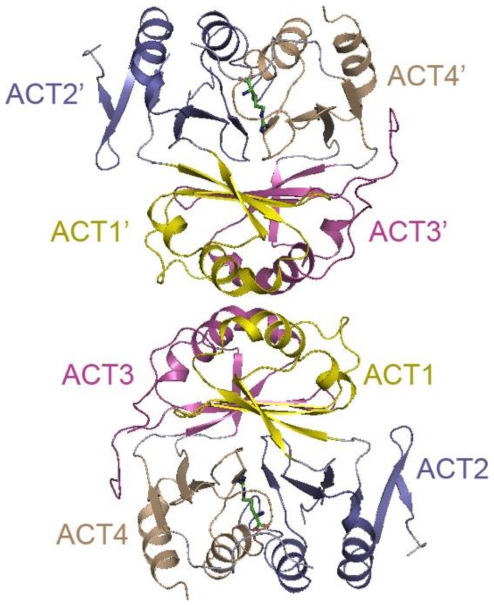
ACT2'
ACT4'
ACT1'
ACT3'
ACT3
ACT1
ACT2
ACT4

B

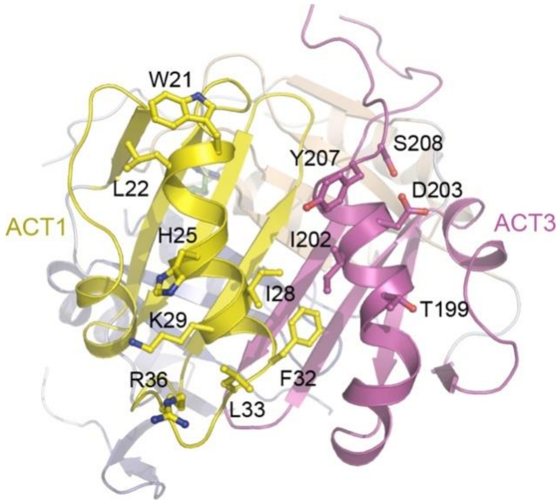
W21
S208
Y207
L22
D203
ACT1
ACT3
H25
I202
I28
K29
T199
R36
F32
L33

Figure S6

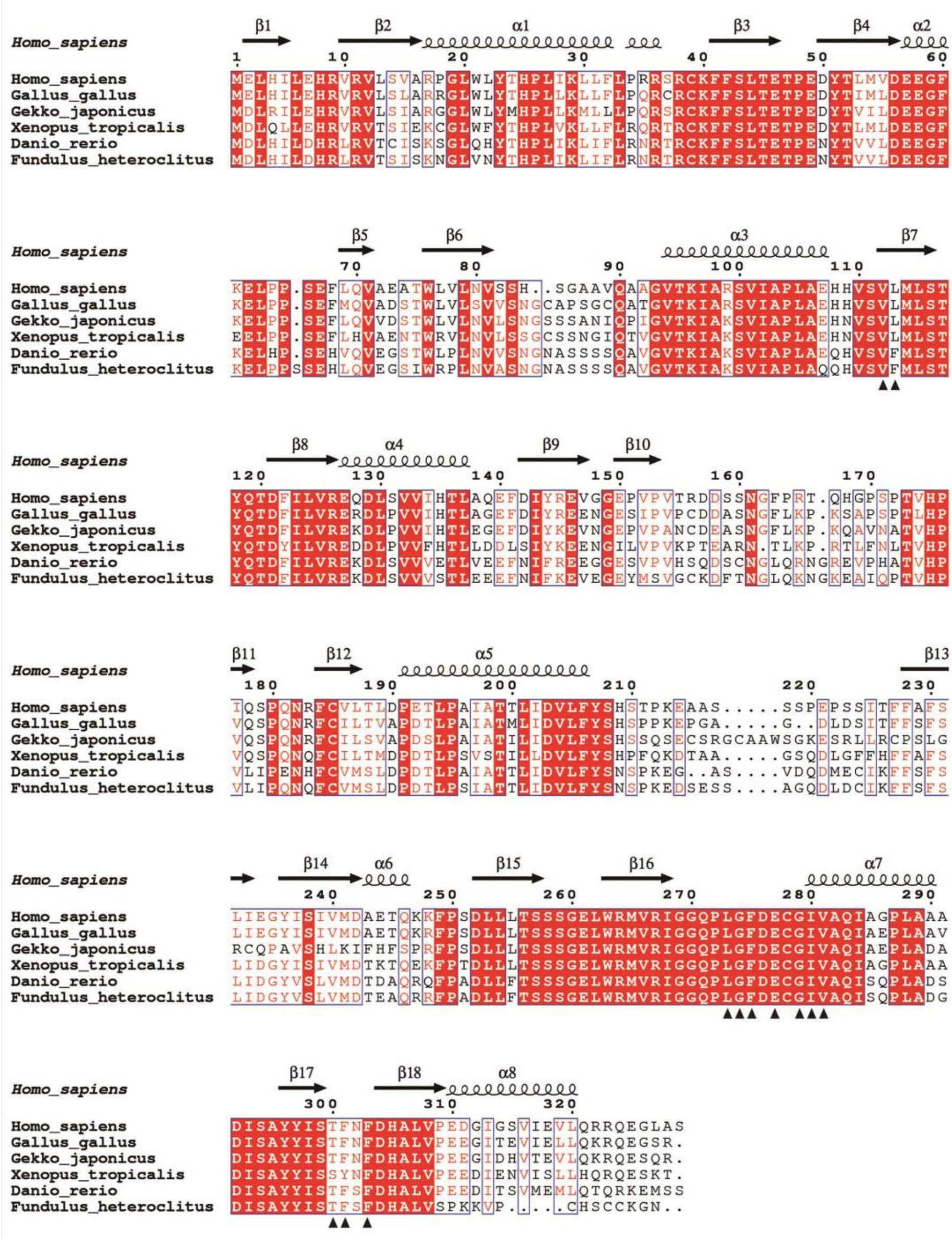

Figure S7

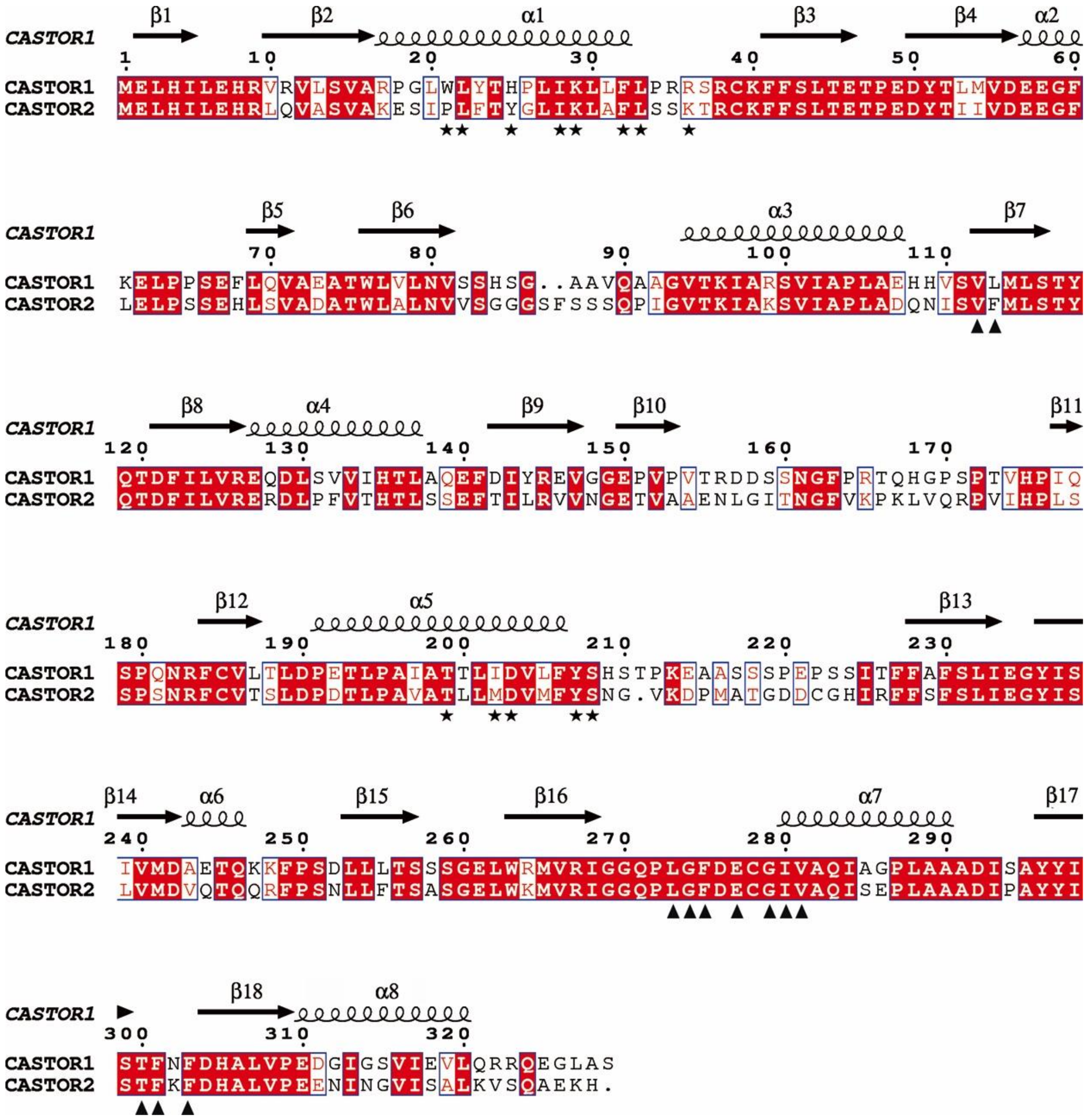

Supplement: Supplementary information [file celldisc201635-s1.pdf]
